# Total Serum Magnesium Levels and Calcium-To-Magnesium Ratio in Sickle Cell Disease

**DOI:** 10.3390/medicina55090547

**Published:** 2019-08-29

**Authors:** Charles Antwi-Boasiako, Yaw A. Kusi-Mensah, Charles Hayfron-Benjamin, Robert Aryee, Gifty Boatemaah Dankwah, Lim Abla Kwawukume, Ebenezer Owusu Darkwa

**Affiliations:** 1 Department of Physiology, School of Biomedical and Allied Health Sciences, University of Ghana, Accra 00233, Ghana; 2 Departments of Anaesthesia, School of Medicine and Dentistry, University of Ghana, Accra 00233, Ghana; 3 Department of Internal Medicine, Korle-Bu Teaching Hospital Accra 00233, Ghana

**Keywords:** magnesium, calcium, homeostasis, cell dehydration, sickle cell disease

## Abstract

*Background and Objectives:* Imbalance of calcium/magnesium ratio could lead to clinical complications in sickle cell disease (SCD). Low levels of magnesium have been associated with sickling, increased polymerization and vaso-occlusion (VOC) in sickle cell due to cell dehydration. The K-Cl cotransport plays a very important role in sickle cell dehydration and is inhibited by significantly increasing levels of magnesium. The study evaluated total serum magnesium levels and computed calcium/magnesium ratio in SCD patients and “healthy” controls. *Materials and Methods:* The study was a case-control cross-sectional one, involving 120 SCD patients (79 Haemoglobin SS (HbSS)and 41 Haemoglobin SC (HbSC)) at the steady state and 48 “healthy” controls. Sera were prepared from whole blood samples (*n* = 168) and total magnesium and calcium measured using a Flame Atomic Absorption Spectrometer (Variant 240FS manufactured by VARIAN Australia Pty Ltd., Melbourne, VIC, Australia). Calcium/magnesium ratios were calculated in patients and the controls. *Results:* The prevalence of hypomagnesemia and hypocalcaemia among the SCD patients was observed to be 39.17% and 52.50% respectively, higher than the controls (4.17% and 22.92%, for hypomagnesemia and hypocalcaemia, respectively). Level of magnesium was significantly lower in the SCD patients compared to their healthy counterparts (*p* = 0.002). The magnesium level was further reduced in the HbSS patients but not significantly different from the HbSC patients (*p* = 0.584). calcium/magnesium ratio was significantly higher in the SCD patients (*p* = 0.031). Although calcium/magnesium ratio was higher in the HbSC patients compared to those with the HbSS genotype, the difference was not significant (*p* = 0.101). *Conclusion:* The study shows that magnesium homeostasis are altered in SCD patients, and their levels are lower in HbSS patients. Although calcium/magnesium ratio is significantly higher in SCD patients compared with controls, there is no significant difference between patients with HbSS and HbSC genotypes. Magnesium supplementation may be required in sickle cell patients.

## 1. Introduction

Sickle cell disease (SCD) is a group of inherited blood disorders characterized by mutations in the gene that encodes the hemoglobin subunit β (HBB), resulting in the sickle Haemoglobin (HbS) allele β^S^. Under deoxygenated conditions, the HbS can undergo polymerization, and form a crescent or sickled shape. Individuals with a homozygous form of the β^S^ are described as having sickle cell anemia (SCA). SCA is the most common form of the SCD characterized by chronic hemolysis and painful vaso-occlusion, resulting in organ dysfunction [1]. Individuals heterozygous for Haemoglobin S and Haemoglobin C (HbSC) also present with several complications and although this is often described as milder form of the disease [2,3,4]. People with HbSS genotype are distinct from those with the HbSC genotype, clinically and hematological [5,6]. Although previous studies have reported that patients with the HbSC genotype in most cases present a milder form of the disease [5], there has also been reports of severe clinical complications in some patients with the HbSC genotype [5,7].

The search for biomarkers of SCD and associated clinical complications in Ghana is crucial, due to the higher prevalence of the disease [8]. Clinical complications of the disease usually progress from mild to severe, with painful vaso-occlusion episodes. Several studies have reported that, vasoconstriction and inflammation significantly contribute to the painful episodes observed in SCD patients. These factors may also account for a possible prolongation of the crises [9,10,11]. The presence of dense erythrocytes, which are thought to play an important role in vaso-occlusion is a distinguishing feature of the sickle erythrocyte [12,13]. These dense erythrocytes are formed as a result of cell dehydration and K^+^ loss through the Ca-activated K channel [14] and the K-Cl cotransport channel [15,16].

Magnesium is a potent vasodilator with anti-inflammatory properties that helps alleviate pain [17,18]. Magnesium does not only inhibit inositol triphosphate calcium channel but modulates several other types of voltage-gated calcium channels [19]. Calcium has also been shown in another studies to stimulate potassium efflux and loss of cell water through calcium-potassium channels in SCD [20].

Reports on the level of magnesium in sickle cell patients have been variable, ranging from low to normal [21,22,23,24]. Previous studies have shown that, magnesium levels are low in sickle cell anemia [21,22], while others reported normal levels [23,24]. In other disease complications, magnesium levels have also been demonstrated to reduce in such patients. Therefore, to monitor disease severity and help in the management of vasculopathies, evaluation of magnesium level, as well as calcium/magnesium ratio are important. Results obtained from a previous clinical trial suggest that, magnesium supplementation can alleviate pain in SCD patients with vaso-occlusive crises [25]. Although studies have reported low levels of magnesium in sickle cell anemia in Europe and some parts of Africa, there exist no reports on magnesium levels in SCD patients in Ghana. The current study therefore reports on level of magnesium and calcium/magnesium ratio in SCD patients with HbSS and HbSC genotypes.

## 2. Materials and Methods

### 2.1. Study Site

The study was conducted at the Korle-Bu Teaching Hospital (KBTH) in the Greater Accra Region. The hospital is the leading tertiary facility in the country, with a bed capacity of about 2000. The sickle cell unit wherein this current study was undertaken forms part of the departments of the facility. The controls were recruited from the Southern Area Blood Centre, Korle-Bu.

### 2.2. Study Design, Subject Recruitment, and Data Collection

The study was a case control cross-sectional one, involving 120 sickle cell patients with HbSS and HbSC genotypes, and 48 HbAA healthy individuals as controls. The patients (at the steady state) were recruited from the sickle cell unit of the KBTH from the period May 2018 to August 2018. Steady state was defined as a period of at least four consecutive weeks with no crises, no blood transfusions during the previous 4 months, and no history of complications such as infection during the previous 4 weeks. Patients with comorbidities such as coronary artery disease, diabetes mellitus, hypertension, renal failure, pregnancy and recent blood transfusion (three months prior to the study) were not included in the study. The cellulose acetate hemoglobin electrophoresis was used to determine hemoglobin genotypes of the study participants. Five milliliters (5 mL) of venous blood sample was collected from each of the study participants into plain tubes for serum magnesium and calcium analysis.

### 2.3. Laboratory Analysis

The blood samples in the tubes were spun in a centrifuge at 2500 rpm for 10 min. The sera were kept at −20 °C before analyses were done. Magnesium and calcium levels were quantified using a Flame Atomic Absorption Spectrometer (Variant 240FS manufactured by VARIAN Australia Pty Ltd., Melbourne, VIC, Australia) with reference ranges of 0.73–1.06 mmol/L and 2.20–2.65 mmol/L respectively. Hypomagnesemia and hypocalcaemia was defined as serum magnesium and serum calcium level below 0.73 mmol/L and 2.20 mmol/L respectively.

### 2.4. Data Analyses

Data from the current study was entered in Microsoft Excel 2010 and analyzed using SPSS version 20. Mean plus or minus standard deviation and frequencies were used to represent nominal data. The student’s t-test was used to compare the means between two groups, and the analysis of variance (ANOVA) was used in the case of more than two groups, and *p*-values < 0.05 were considered significant.

### 2.5. Ethical Statement

Ethical approval for the study was sought from the Ethical and Protocol Review Committee of the College of Health Sciences, University of Ghana on 26 April 2018 with protocol identification number Et/M.8-P2.13/2017-2018. Blood samples and demographic data were obtained from study participants following their consent to partake in the study.

## 3. Results

### 3.1. Demographical and Clinical Characteristics of the Study Participants

The 168 study participants recruited into the study were made up of 120 SCD patients (79 hemoglobin SS and 41 hemoglobin SC genotypes and 48 HbAA controls). The mean age of SCD patients was 27.66 ± 10.57. Females were slightly higher than males in the SCD patients but not in the controls. There were no significant differences in gender among the study participants (*p* = 0.440). The general characteristics of the study participants are shown in Table 1.

### 3.2. Serum Levels of Magnesium and Calcium in SCD Patients and the Controls

The mean serum levels of both magnesium and calcium were compared between SCD patients and the apparently healthy controls (without SCD). The level of magnesium was significantly higher (*p* = 0.002) in the control group. Mean serum level of calcium was also found to be significantly higher (*p* = 0.012) in the control group compared to the SCD patients. When calcium to magnesium ratio was computed, it was observed that, SCD patients had significantly higher (*p* = 0.031) calcium to magnesium ratio compared to their healthy counterparts. Serum levels of magnesium and calcium of the study participants are shown in Table 2.

### 3.3. Serum Magnesium and Calcium Levels in Patients with HbSS and HbSC Genotypes

Serum levels of magnesium and calcium was further evaluated in the HbSS and HbSC patients. The difference between HbSS patients (*n* = 79) and HbSC patients (*n* = 41) in the levels of magnesium was not significant (*p* = 0.584). A similar trend was observed in patients with HbSS and HbSC when the serum levels of calcium in these two study subjects was compared (*p* = 0.167). There was no significant difference in the level of calcium to magnesium ratio between the two study patients (HbSS SCD versus HbSC SCD) (*p* = 0.101). Levels of serum magnesium and calcium in HbSS and HbSC patients are shown in Table 3.

There was a wide range of serum magnesium and calcium in the SCD patients as well as the controls (Table 4). However, from the reference range used in our laboratory method of sample analysis, a high prevalence of hypomagnesemia and hypocalcemia was noted among the sickle cell patients compared to the controls (Table 5).

## 4. Discussion

Magnesium plays a very significant role in bone and sugar metabolism, arterial blood pressure, immune system function, heart rhythm and SCD [18,26,27,28,29,30]. The lower levels of magnesium in the SCD patients compared with their “healthy” counterparts (HbAA) suggest in part that, increased hemolysis due to HbS polymerization observed in SCD and associated complications rapidly depletes magnesium [31,32]. The hemoglobin S, found in the erythrocyte of sickle cell patients auto-polymerize faster than the normal hemoglobin [33], which may lead to increased dehydration [34] and lower levels of magnesium.

In sickle cell, the abnormally high red cell permeability and loss of potassium favors higher propensity for red cell dehydration. The K-Cl co-transport is one of the pathways through which potassium are lost; which is abnormally activated by low intracellular magnesium [31]. In principle, K^+^ and Cl^−^ ions are rapidly and irreversibly lost with a very significant amount of water following, as a result of osmosis [35]. Thus, higher levels of intracellular magnesium could block this pathway, and reduce dehydration as well as sickling in SCD patients [34]. A significantly high prevalence of hypomagnesemia and hypocalcemia in SCD patients was found in this study compared with the controls. In line with similar studies [21,22,32], magnesium levels were observed to be reduced in sickle cell patients. Magnesium supplementation in these patients could help regulate the channels through which K^+^ are lost; thereby reducing painful episodes frequently encountered by patients with sickle cell [18,36]. However, results (supported by low quality evidence) from a recent study by Than et al. [37] revealed that neither oral nor intravenous magnesium supplementation has an effect on treating painful episodes in sickle cell patients. Yet, in this review, none of the study involved was from sub-Saharan Africa. It would been interesting to know how a study from the sub-region can influence these particular findings. There is a paucity of literature which establishes a possible reduction of red cell dehydration in sickle cell patients, following magnesium supplementation (whether oral or intravenous) in sub-Saharan Africa. Based on our findings, the authors will propose a possible multicenter randomized controlled study, which focuses on magnesium supplementation and its effect on painful crises in sickle cell patients in the sub-region.

It was observed that patients with the HbSS genotype, characterized by chronic hemolysis [1], had reduced levels of magnesium compared with the HbSC individuals, although not significant. In line with the current study, a previous study observed that, homozygous sickle cell patients (SS) characterized by painful vaso-occlusive crises, intense hemolysis and anemia frequently encountered by sickle cell patients with the HbSS genotype [38,39,40], generally have low levels of magnesium. This could possibly lead to a higher efflux via the Gardos channel and thus, increase cell dehydration [15]. Findings from this current study suggest in part that, there may be similar underlying conditions in the two SCD genotypes studied (HbSS and HbSC), which resulted in the comparable magnesium levels.

Normal serum-magnesium values do not consistently reflect intracellular levels [41]. As serum magnesium decreases, the body pulls magnesium out of red blood cells in the bone and tissue, in order to compensate for that decrease. Decreased red blood cell (RBC) magnesium levels have been reported despite normal serum magnesium levels due to the increased activity of the Na/Mg exchanger in Mg-loaded sickle erythrocytes [31]. However, there are conflicting findings regarding magnesium and SCD. The authors [42] in one study reported a low serum magnesium levels in SCD and in a separate study [43] reported low RBC magnesium to have a negative correlation with serum magnesium levels in SCD. Another study [44] also noted reduced serum magnesium but elevated RBC magnesium levels in SCD. Yousif and colleagues [22] on the other hand, reported both a significant reduction in serum and RBC magnesium levels but noted a significant negative association between the RBC magnesium and the frequency of vaso-occlusive crisis (VOC) in SCD patients. However, in Nigeria [45] a significant negative association was found between serum magnesium levels and frequency of VOC in SCD patients. However, in the study conducted in Nigeria, RBC magnesium level was not measured. In addition to RBC dehydration and therefore complications in SCD patients, magnesium may also cause vasodilatation through nitric oxide release and inhibition of calcium in smooth muscle. Magnesium prolongs clotting time, hence a reduction in vaso-occlusion [25].

In our current study, we measured serum magnesium levels but did not measure RBC magnesium levels. From the conflicting literature above it is somehow obvious that most studies that reported low RBC magnesium levels in SCD patients either reported low or normal serum magnesium levels. Therefore, our finding of a significant reduction in serum magnesium levels in SCD patients in Ghana is meaningful. However, we will recommend a further study to ascertain RBC magnesium levels in SCD patients so as to confirm its association with serum magnesium and other electrolytes. The conflicting results could also be due to differences in analytical methods used in determining serum and RBC magnesium levels as well as possible differences in sample handling. Other limitations of the study include assessing magnesium status of patients only in the steady state and not during VOC or other SCD associated complications. Future studies on the role and mechanism of magnesium at the tissue level will obviously enhance better understanding and development of therapeutic interventions.

Significantly higher mean serum calcium/magnesium ratio was found in the SCD patients compared with the controls in the present study. High serum calcium/magnesium ratio observed in the SCD patients suggests that the Gardos channel could be activated, leading to K^+^ loss and dehydration [32]. Thus, calcium/magnesium ratio could help predict the extent of dehydration in SCD. The difference in mean serum calcium/magnesium ratio between HbSS and HbSC patients was however, not significant. Low magnesium levels and high mean serum calcium/magnesium could lead to several complications of the SCD such as VOC [32], as observed in other vasculopathies [46,47,48]. High calcium/magnesium and low levels of magnesium have been proposed to cause cell dehydration, by the activation of the Gardos channel and the K-Cl co-transport [31,32]. Therefore, it was important to determine the ratio of calcium/magnesium in the study subjects. This study provides an important information on magnesium levels and calcium/magnesium ratio, implicated in the pathophysiology of SCD. Knowledge of calcium/magnesium ratio may partly reveal the extent of cell dehydration and could help in the management of SCD crises. The main limitation of the study was the smaller sample size of all the genotypes.

## 5. Conclusions

The prevalence of hypomagnesemia and hypocalcemia among the SCD patients was higher compared to the controls. SCD patients in this study had low serum levels of magnesium compared to the controls. The mean serum magnesium level in the HbSS patients was not significantly different from the HbSC group. Mean serum calcium/magnesium ratio was higher in the SCD patients. Although mean serum calcium/magnesium ratio was elevated in the HbSC patients compared to those with HbSS genotype; the difference was not significant.

## Figures and Tables

**Table 1:** Demographic characteristics of the study participants.

Variable	HbSS (*n* = 79)	HbSC (*n* = 41)	HbAA (*n* = 48)	*p*-Value
Age (mean ± SD)	27.08 ± 9.92	28.82 ± 11.80	31.67 ± 9.48	0.058
Sex *n* (%)				
Male	32 (42.1)	19 (25.0)	25 (32.9)	0.440
Female	47 (51.1)	22 (23.9)	23 (25.0)	
Ethnicity *n* (%)				
Akan	37 (46.8)	17 (41.5)	23 (47.9)	
Ewe	11 (13.9)	11 (26.8)	10 (20.8)	
Ga-Adangbe	21 (26.6)	7 (17.1)	8 (16.7)	0.605
Mole/Dagomba	1 (1.3)	2 (4.9)	3 (6.3)	
Guan/Gume	1 (1.3)	0 (0.0)	1 (2.0)	
Others	8 (10.1)	4 (9.8)	3 (6.3)	

HbSS: Haemoglobin SS, HbSC: haemoglobin SC, HbAA: Normal haemoglobin, *p* < 0.05 is significant.

**Table 2:** Serum magnesium and calcium levels in the study participants.

Variable	SCD Patients (*n* = 120)	Controls (*n* = 48)	*p*-Value
Magnesium (mmol/L)	0.80 ± 0.24	0.90 ± 0.11	0.002
Calcium (mmol/L)	2.11 ± 0.38	2.28 ± 0.53	0.012
Calcium/Magnesium ratio	2.80 ± 0.72	2.54 ± 0.89	0.031

SCD: Sickle cell disease, *p* < 0.05 is significant.

**Table 3:** Serum magnesium and calcium levels in HbSS and HbSC patients.

Variable	HbSS (*n* = 79)	HbSC (*n* = 41)	*p*-Value
Magnesium (mmol/L)	0.79 ± 0.25	0.82 ± 0.21	0.584
Calcium (mmol/L)	2.07 ± 0.39	2.17 ± 0.36	0.167
Calcium/Magnesium ratio	2.79 ± 0.71	2.82 ± 0.76	0.101

*p* < 0.05 is significant.

**Table 4:** Range of serum magnesium and Calcium levels among the study participants.

Variable	HbSS (*n* = 79)	HbSC (*n* = 41)	HbAA (*n* = 48)
Serum magnesium (mmol/L)	(0.22–1.49)	(0.32–1.12)	(0.30–1.06)
Serum calcium (mmol/L)	(0.73–2.59)	(0.94–2.71)	(0.17–2.82)

**Table 5:** Prevalence of hypomagnesemia and hypocalcemia among the sickle cell disease (SCD) patients and the controls.

Variable	SCD (*n* = 120)	HbAA (*n* = 48)	χ-Square, *p*-Value	HbSS (*n* = 79)	HbSC (*n* = 41)	χ-Square, *p*-Value
Magnesium	47 (39.17%)	2 (4.17%)	20.21, <0.001	35 (44.30%)	11 (26.83%)	3.46, 0.063
Calcium	63 (52.50%)	11 (22.92%)	12.10, 0.001	44 (55.70%)	19 (46.34%)	0.94, 0.332

χ-Square: Chi-square test, *p* < 0.05 is significant.
